# Supplementary figures and images for: Tryptamine accumulation caused by deletion of MrMao-1 in Metarhizium genome significantly enhances insecticidal virulence
Source: PLoS Genet. 2020 Apr 9;16(4):e1008675. doi: 10.1371/journal.pgen.1008675 (PMC7173932; doi:10.1371/journal.pgen.1008675)

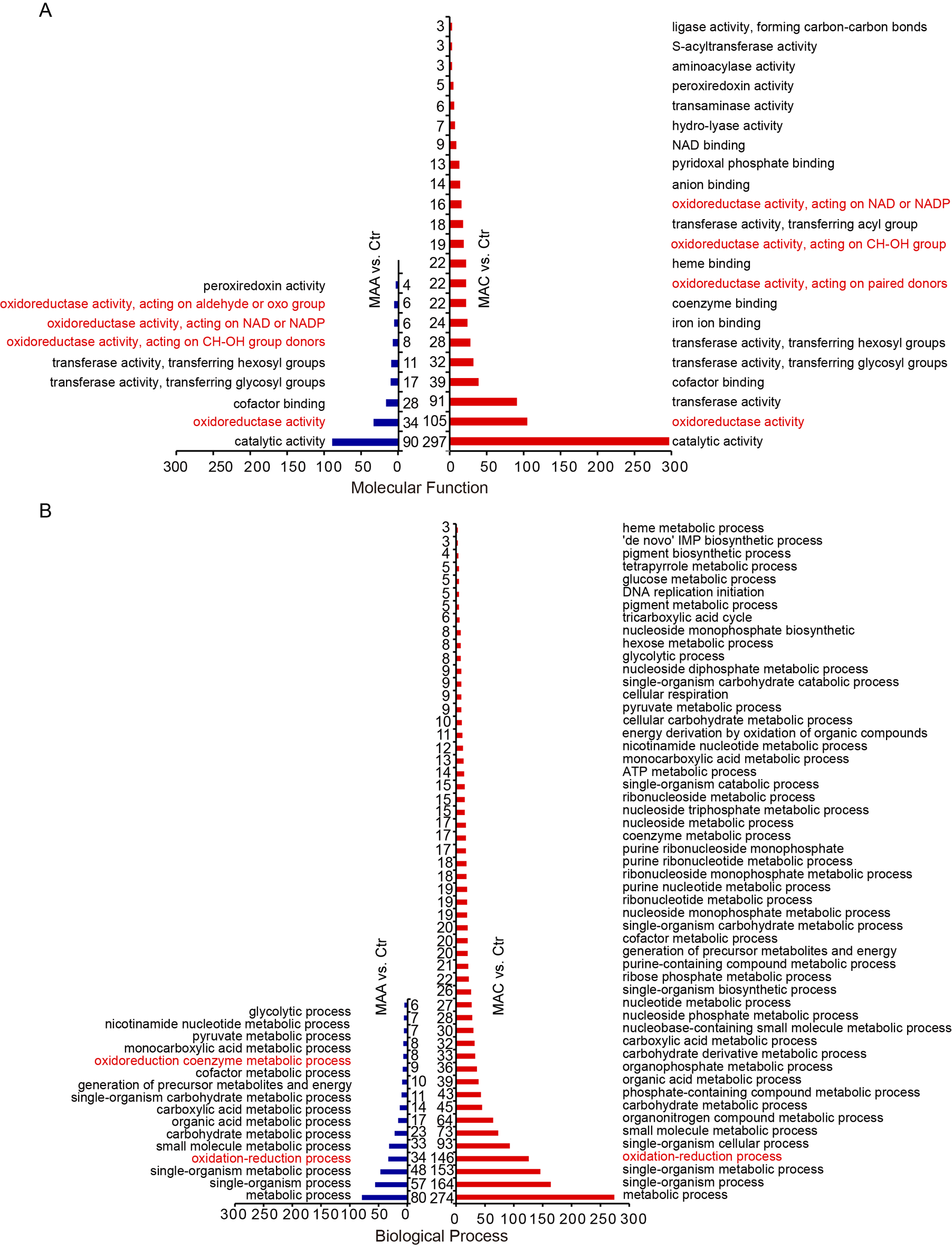

Supplement: S1 Fig — (A) Class of biological process in MAA- and MAC-infected locusts. (B) Class of molecular function in MAA- and MAC-infected locusts. (TIF) [file pgen.1008675.s001.tif]

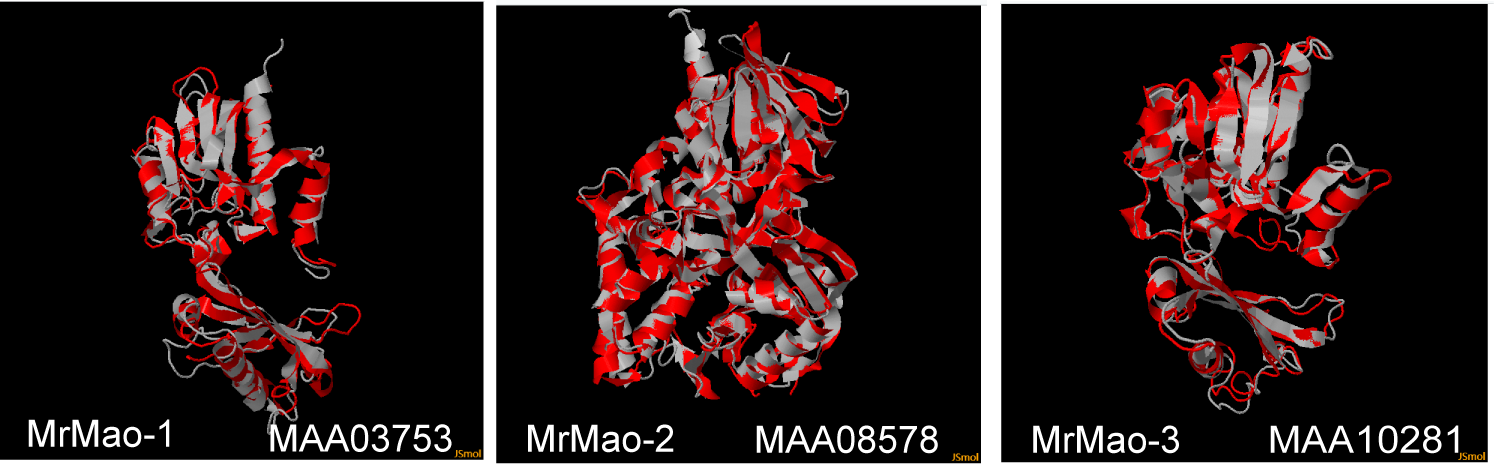

Supplement: S2 Fig — A 3-D structure of MrMAO proteins was constructed by Protein Homology/analogY Recognition Engine V 2.0 (Phyre2). The protein structures of MrMAO were found to be highly similar to human MAOB (PDB number: 1S3B). The root mean square deviations of MrMAO-1, MrMAO-2, and MrMAO-3 calculated by FATCAT simulation (http://fatcat.burnham.org/fatcat/) were 2.38, 1.46, and 2.76, respectively. The red structure is the human MAOB, and the gray structure is MrMAO. (TIF) [file pgen.1008675.s002.tif]
