# Supplementary material for: Tryptamine accumulation caused by deletion of MrMao-1 in Metarhizium genome significantly enhances insecticidal virulence
Source: PLoS Genet. 2020 Apr 9;16(4):e1008675. doi: 10.1371/journal.pgen.1008675 (PMC7173932; doi:10.1371/journal.pgen.1008675)
Supplement: S1 Table — (DOCX) [file pgen.1008675.s003.docx]

**S1 Table Top DEGs uniquely induced by MAC infection.**

| GeneID | Annotation | LogFC (MAC/CTR) | LogFC (MAC/MAA) |
| --- | --- | --- | --- |
| LOCMI15816 | V-type proton ATPase 16 kDa proteolipid subunit | 0.035872313 | 7.572348 |
| LOCMI04373 | defensin | 0.109773526 | 6.015885 |
| LOCMI13596 | Multidrug resistance-associated protein 4 | 0.464481878 | 5.766647 |
| LOCMI04741 | Multidrug resistance protein homolog 49 | 1.026670548 | 4.988172 |
| LOCMI07113 | Multidrug resistance-associated protein 4 | 0.640431234 | 4.916825 |
| LOCMI03329 | Glucose dehydrogenase [FAD, quinone] | 0.486579984 | 4.176769 |
| LOCMI14068 | Serine proteinase stubble | 0.402106392 | 4.128507 |
| LOCMI05806 | Aspartate aminotransferase, cytoplasmic | 0.540926209 | 3.999484 |
| LOCMI15614 | carboxylesterase-like protein | 0.590401169 | 3.289207 |
| LOCMI08097 | Sialin | 0.583989622 | 2.996729 |
| LOCMI17245 | hemocyanin subunit type 2 | 1.735239088 | 2.970014 |
| LOCMI16443 | Cysteine sulfinic acid decarboxylase | 1.138390281 | 2.680218 |
| LOCMI05769 | Zinc transporter ZIP14 | 0.243635601 | 2.656126 |
| LOCMI15742 | Glutamate receptor ionotropic, delta-1 | 0.960178895 | 2.636313 |
| LOCMI03487 | Protein big brother | 0.214512867 | 2.536715 |
| LOCMI12274 | LOCMI12274 | 0.036572643 | 2.489556 |
| LOCMI04376 | Myelin regulatory factor | 1.739236911 | 2.44548 |
| LOCMI16349 | Serine protease snake | 0.00305476 | 2.381169 |
| LOCMI07135 | Inositol-pentakisphosphate 2-kinase | 0.54331609 | 2.333241 |
| LOCMI09002 | Peptidylglycine alpha-hydroxylating monooxygenase | 0.100789296 | 2.235917 |
| LOCMI05010 | Inverted formin-2 | 0.789926596 | 2.135443 |
| LOCMI15659 | Ejaculatory bulb-specific protein 3 | 0.16483275 | 2.123502 |
| LOCMI00587 | Probable multidrug resistance-associated protein lethal(2)03659 | 0.070988724 | 2.081247 |
| LOCMI02859 | Inter-alpha-trypsin inhibitor heavy chain H4 | 0.897558987 | 2.022304 |
| LOCMI02438 | Endoglucanase E-4 | 0.260244346 | 1.957507 |
| LOCMI13201 | UTP--glucose-1-phosphate uridylyltransferase | 0.885487284 | 1.93891 |
| LOCMI11262 | Vanin-like protein 2 | 0.084917407 | 1.93712 |
| LOCMI04753 | Vesicular glutamate transporter 2 | 0.062339409 | 1.896725 |
| LOCMI01596 | Probable multidrug resistance-associated protein lethal(2)03659 | 0.398099042 | 1.880114 |
| LOCMI01675 | serine proteinase | 0.835482633 | 1.85174 |
| LOCMI00556 | Multidrug resistance-associated protein 4 | 0.319263449 | 1.843768 |
| LOCMI07086 | PREDICTED: venom protease-like | 0.291112128 | 1.793325 |
| LOCMI15541 | Probable multidrug resistance-associated protein lethal(2)03659 | 1.030356255 | 1.719369 |
| LOCMI02122 | Inter-alpha-trypsin inhibitor heavy chain H4 | 0.982412561 | 1.706743 |
| LOCMI15210 | Aquaporin AQPAe.a | 0.996915939 | 1.686203 |
| LOCMI11276 | Calcium release-activated calcium channel protein 1 | 0.124625232 | 1.674981 |
| LOCMI16679 | Proto-oncogene tyrosine-protein kinase receptor Ret | 1.20099579 | 1.6352 |
| LOCMI05263 | Laccase-4 | 0.886617304 | 1.606196 |
| LOCMI00232 | Ejaculatory bulb-specific protein 3 | 0.134707635 | 1.546501 |
| LOCMI09778 | Enteropeptidase | 0.183208481 | 1.52181 |
| LOCMI16088 | Phospholipase A2D | 0.697643498 | 1.516907 |
| LOCMI12006 | Putative uncharacterized protein | 0.572644945 | 1.495657 |
| LOCMI16091 | Glucose-6-phosphate 1-epimerase | 0.230988473 | 1.484679 |
| LOCMI12621 | Cuticlin-1 | 1.008424864 | 1.479384 |
| LOCMI08674 | Protein FAM151B | 0.101299668 | 1.468056 |
| LOCMI08619 | Cystinosin homolog | 0.139991149 | 1.457212 |
| LOCMI03467 | Nebulette | 0.02588182 | 1.416783 |
| LOCMI09644 | AGAP011666-PA | 0.030920479 | 1.391713 |
| LOCMI05778 | Protein-glutamine gamma-glutamyltransferase | 0.608336065 | 1.3425 |
| LOCMI10455 | ATP-binding cassette sub-family G member 1 | 0.053147569 | 1.324467 |
| LOCMI03127 | E3 ubiquitin-protein ligase SlrP | 0.183298511 | 1.280683 |
| LOCMI06174 | Tyrosine-protein phosphatase 10D | 0.410791695 | 1.264164 |
| LOCMI16405 | NF-kappa-B inhibitor cactus | 0.160956695 | 1.210401 |
| LOCMI15898 | Peroxisomal acyl-coenzyme A oxidase 3 | 0.262138885 | 1.175395 |
| LOCMI16463 | Protein decapentaplegic | 0.055819743 | 1.174135 |
| LOCMI11743 | HsjCib isoform1 | 0.100862079 | 1.118544 |
| LOCMI00101 | Tyrosine-protein phosphatase 10D | 0.265939413 | 1.085334 |
| LOCMI14056 | Sushi, nidogen and EGF-like domain-containing protein 1, partial | 0.908337017 | 1.078663 |
| LOCMI16832 | Protein kinase C iota type | 0.333808243 | 1.07642 |
| LOCMI09089 | Dual 3',5'-cyclic-AMP and -GMP phosphodiesterase 11 | 0.108823507 | 1.003738 |
| LOCMI07914 | Sodium- and chloride-dependent GABA transporter 1 | 0.311487692 | 0.993558 |
| LOCMI15833 | Hexokinase type 2 | 0.443161104 | 0.972341 |
| LOCMI11691 | MAGUK p55 subfamily member 6 | 0.295230242 | 0.851025 |
| LOCMI13441 | Uncharacterized peptidase C1-like protein F26E4.3 | 0.513790474 | 0.803598 |
| LOCMI05746 | Regulator of G-protein signaling loco | 0.248116296 | 0.756662 |
| LOCMI13623 | UDP-glucose 4-epimerase | 0.078663922 | 0.743461 |
| LOCMI01884 | Asparagine--tRNA ligase, cytoplasmic | 0.015468969 | 0.727412 |
