# Supplementary material for: Tryptamine accumulation caused by deletion of MrMao-1 in Metarhizium genome significantly enhances insecticidal virulence
Source: PLoS Genet. 2020 Apr 9;16(4):e1008675. doi: 10.1371/journal.pgen.1008675 (PMC7173932; doi:10.1371/journal.pgen.1008675)
Supplement: S2 Table — (DOCX) [file pgen.1008675.s004.docx]

**S2 Table Calculation of lethal dosage of tryptamine to locusts by Probit methods.**

| Probabolity | | 95% confidence limit | | |  | 95% confidence limit of log | | | | | | |  |
| --- | --- | --- | --- | --- | --- | --- | --- | --- | --- | --- | --- | --- | --- |
|  | Calculation | | Lower limit | | | Upper limit | Calculation | | Lower limit | | Upper limit | | |
| 0.01 | 0.005 | | 0 | 0.059 | | -2.261 | | -4.703 | | -1.23 | |  |  |
| 0.02 | 0.012 | | 0 | 0.107 | | -1.905 | | -4.09 | | -0.969 | | |  |
| 0.03 | 0.021 | | 0 | 0.157 | | -1.679 | | -3.703 | | -0.803 | | |  |
| 0.04 | 0.031 | | 0 | 0.211 | | -1.509 | | -3.412 | | -0.676 | | |  |
| 0.05 | 0.043 | | 0.001 | 0.267 | | -1.37 | | -3.177 | | -0.573 | | |  |
| 0.06 | 0.056 | | 0.001 | 0.328 | | -1.253 | | -2.977 | | -0.484 | | |  |
| 0.07 | 0.071 | | 0.002 | 0.392 | | -1.149 | | -2.802 | | -0.406 | | |  |
| 0.08 | 0.088 | | 0.002 | 0.462 | | -1.057 | | -2.646 | | -0.336 | | |  |
| 0.09 | 0.106 | | 0.003 | 0.536 | | -0.973 | | -2.505 | | -0.271 | | |  |
| 0.1 | 0.127 | | 0.004 | 0.615 | | -0.896 | | -2.375 | | -0.211 | | |  |
| 0.15 | 0.266 | | 0.014 | 1.099 | | -0.575 | | -1.842 | | 0.041 | | |  |
| 0.2 | 0.478 | | 0.037 | 1.773 | | -0.321 | | -1.426 | | 0.249 | | |  |
| 0.25 | 0.79 | | 0.084 | 2.72 | | -0.102 | | -1.077 | | 0.435 | | |  |
| 0.3 | 1.242 | | 0.17 | 4.068 | | 0.094 | | -0.771 | | 0.609 | | |  |
| 0.35 | 1.887 | | 0.319 | 6.031 | | 0.276 | | -0.496 | | 0.78 | | |  |
| 0.4 | 2.808 | | 0.568 | 8.968 | | 0.448 | | -0.246 | | 0.953 | | |  |
| 0.45 | 4.123 | | 0.966 | 13.507 | | 0.615 | | -0.015 | | 1.131 | | |  |
| 0.5 | 6.019 | | 1.585 | 20.795 | | 0.779 | | 0.2 | | 1.318 | | |  |
| 0.55 | 8.785 | | 2.524 | 32.997 | | 0.944 | | 0.402 | | 1.518 | | |  |
| 0.6 | 12.901 | | 3.924 | 54.418 | | 1.111 | | 0.594 | | 1.736 | | |  |
| 0.65 | 19.193 | | 6.005 | 94.119 | | 1.283 | | 0.779 | | 1.974 | | |  |
| 0.7 | 29.169 | | 9.127 | 172.689 | | 1.465 | | 0.96 | | 2.237 | | |  |
| 0.75 | 45.825 | | 13.946 | 341.857 | | 1.661 | | 1.144 | | 2.534 | | |  |
| 0.8 | 75.781 | | 21.776 | 750.921 | | 1.88 | | 1.338 | | 2.876 | | |  |
| 0.85 | 136.207 | | 35.671 | 1928.371 | | 2.134 | | 1.552 | | 3.285 | | |  |
| 0.9 | 284.832 | | 64.575 | 6493.481 | | 2.455 | | 1.81 | | 3.812 | | |  |
| 0.91 | 340.387 | | 74.276 | 8735.836 | | 2.532 | | 1.871 | | 3.941 | | |  |
| 0.92 | 413.085 | | 86.369 | 12072.01 | | 2.616 | | 1.936 | | 4.082 | | |  |
| 0.93 | 511.065 | | 101.819 | 17250.57 | | 2.708 | | 2.008 | | 4.237 | | |  |
| 0.94 | 648.206 | | 122.187 | 25737.31 | | 2.812 | | 2.087 | | 4.411 | | |  |
| 0.95 | 850.072 | | 150.191 | 40685.88 | | 2.929 | | 2.177 | | 4.609 | | |  |
| 0.96 | 1168.928 | | 191.029 | 69813.54 | | 3.068 | | 2.281 | | 4.844 | | |  |
| 0.97 | 1729.217 | | 256.126 | 135918.7 | | 3.238 | | 2.408 | | 5.133 | | |  |
| 0.98 | 2910.159 | | 376.929 | 330672.2 | | 3.464 | | 2.576 | | 5.519 | | |  |
| 0.99 | 6610.389 | | 688.733 | 1351013 | | 3.82 | | 2.838 | | 6.131 | | |  |
