# Supplementary material for: Tryptamine accumulation caused by deletion of MrMao-1 in Metarhizium genome significantly enhances insecticidal virulence
Source: PLoS Genet. 2020 Apr 9;16(4):e1008675. doi: 10.1371/journal.pgen.1008675 (PMC7173932; doi:10.1371/journal.pgen.1008675)
Supplement: S3 Table — (DOCX) [file pgen.1008675.s005.docx]

**S3 Table LT50 data of six insect species after infection of WT and *Δmao* strain of MAA.**

| Species | LT_50_-WT(days) | LT_50_-Δ*mao* (days) | Pvlaue |
| --- | --- | --- | --- |
| *Locusta migratoria* | 7.33±0.46 | 5.92 ±0.49 | 0.027 |
| *Tenebrio molitor* | 8.63±0.49 | 7.05 ±0.462 | 0.029 |
| *Acyrthosiphon pisum* | 5.11±0.23 | 4.02 ±0.23 | <0.01 |
| *Aedes aegypti* | 6.25±0.312 | 5.358±0.28 | <0.01 |
| *Bombyx mori* | 3.32±0.19 | 2.29±0.19 | <0.01 |
| *Periplaneta americana* | 14.58±0.68 | 12.28 ±0.76 | 0.05 |
