# Supplementary material for: Tryptamine accumulation caused by deletion of MrMao-1 in Metarhizium genome significantly enhances insecticidal virulence
Source: PLoS Genet. 2020 Apr 9;16(4):e1008675. doi: 10.1371/journal.pgen.1008675 (PMC7173932; doi:10.1371/journal.pgen.1008675)
Supplement: S4 Table — (DOCX) [file pgen.1008675.s006.docx]

**S4 Table All PCR primers used in the experiments.**

| Name | Sequence | Remark | Accession numbers |
| --- | --- | --- | --- |
| Mr03753-1U | ATTCCTGCAGCCCGGGATGGCGACAACCCAAATC | For knockout | MAA03753 |
| Mr03753-1L | CGACGGATCCCCCGGGGTGTCAACCCTCGTTCTATT |  |  |
| Mr03753-2U | GATCTGATGAACTAGTGTTTCGGAACATTCACTTTG |  |  |
| Mr03753-2L | CCGCTCTAGAACTAGTCGGGCAAGATTCCGTTCGT |  |  |
| mr03753F | CAAGCTGGGCTACTACTCA | For qPCR and RT-PCR |  |
| mr03753R | TCTCCGTCCCAACAAAGTG |  |  |
| mr08578F | AAAGGCAACTGGGCACAAC | For qPCR and RT-PCR | MAA08578 |
| mr08578R | CGGCAATGACCTTTCCCAC |  |  |
| Mr08578-1U | ATTCCTGCAGCCCGGGGCCGTTGAACTGATGCCG | For knockout |  |
| Mr08578-1L | CGACGGATCCCCCGGGCCAGGAATGCAGCGTTTGG |  |  |
| Mr08578-2U | GATCTGATGAACTAGTCATCATCATGGGCTTCCTTG |  |  |
| Mr08578-2L | CCGCTCTAGAACTAGTGGTAGGACGGAGACTTGC |  |  |
| mr10281F | CGATCACGTTTGCCGAATAC | For qPCR and RT-PCR | MAA10281 |
| mr10281R | GAGGCTTTCGACCGTTGTC |  |  |
| Mr10281-1U | ATTCCTGCAGCCCGGGATGTCGCCCACGGTTCAGG | For knockout |  |
| Mr10281-1L | CGACGGATCCCCCGGGGTCTCCATACACCGTAACAA |  |  |
| Mr10281-2U | GATCTGATGAACTAGTAAATGCTCCTGGCGAGTTC |  |  |
| Mr10281-2L | CCGCTCTAGAACTAGTGAGAGTGCGGTCTTCTCAT |  |  |
| Tub-F | GATCTTGAACCTGGCACCAT | For RT-PCR | MAA02081 |
| Tub-R | CCATGAAGAAGTGCAGACGA |  |  |
| cyp4c3-F | CCTGGGTGTCAGCATAGTG | For qPCR | MH393902 |
| cyp4c3-R | GTTCCCATAGCAGTCTCAC |  |  |
| cyp4c1a-F | CAATGAAGCCGAAGACAATG | For qPCR | MH393903 |
| cyp4c1a-R | GTAGGACTTCGCTGGGTG |  |  |
| cyp6k1a-F | TGGCGGACTTCTTGAGGAT | For qPCR | MH393906 |
| cyp6k1a-R | GCTTCAGTACCTCGTCTATC |  |  |
| cyp6k1c-F | AGAAGGCAGGGATTTCGTC | For qPCR | MH393908 |
| cyp6k1c-R | GAGTAGCGGGCAATCAGC |  |  |
| rp49-F | CGTAAACCGAAGGGAATTGA | For qPCR |  |
| rp49-R | GAAGAAACTGCATGGGCAAT |  |  |
| stubble-1-F | GCTGGTCGTTCAAGAGGC | For qPCR | MH393896 |
| stubble-1-R | ACCTGCGGAATCAACCTTTT |  |  |
| cactus-F | GTACCAGCACCATGTCCC | For qPCR | MH393899 |
| cactus-R | ACATCTCAGCGTCATCGTC |  |  |
| easter-F | ACTCCGCTTTCATCAGACC | For qPCR | MH393895 |
| easter-R | CAAATGACACGATTCCGATG |  |  |
| lectin-F | ACTTGCTCGCCTGGAACG | For qPCR | MH393897 |
| lectin-R | CTGCCTCTTCCACGACCT |  |  |
| deFensin-F | GTCGTCTTCGCGTCTGTC | For qPCR | MH393898 |
| deFensin-R | CGCCCTTGTAGCCCTTGT |  |  |
| LmMao-a-F | GTTGTCCCATTCAATACCTC | For qPCR | MK125031 |
| LmMao-a-R | GTATGCAATGTAGGCAACTC |  |  |
| LmMao-b-F | TCAGACAGGGTTGGTGGC | For qPCR | MK125030 |
| LmMao -b-R | AGCTCACAGCCCAGTTCTT |  |  |
| cyp6j1-F | GCCGCACCAGTTCATTCC | For qPCR | MK125032 |
| cyp6j1-R | GAAGCTGAGCACCAGAGG |  |  |
| LmTdc-F | TGCGAGCGAGTGCATCCT | For qPCR | MK140601 |
| LmTdc-R | GGCAGATGAAGGCGTTGC |  |  |
| LmAhR-F | GGCGACACTGGAGATGAC |  | MH393891 |
| LmAhR-R | AAGGAGGGCTTGTAGTG | For qPCR |  |
| dsAhR-F | TAATACGACTCACTATAGGCAGGTTTAAAAGCAAAGAGGG | For RNAi |  |
| dsAhR-R | TAATACGACTCACTATAGG TCCACCAAGCCCAGCATC |  |  |
