# Supplementary material for: Tryptamine accumulation caused by deletion of MrMao-1 in Metarhizium genome significantly enhances insecticidal virulence
Source: PLoS Genet. 2020 Apr 9;16(4):e1008675. doi: 10.1371/journal.pgen.1008675 (PMC7173932; doi:10.1371/journal.pgen.1008675)
Supplement: Attachment — Submitted filename: Response to reviewers-final.docx [file pgen.1008675.s007.docx]

COMMENTS FROM REVIEWER#1

Reviewer #1: Well written and well thought out experimental design. The only thing I would suggest is to check the grammar and syntax throughout the manuscript.

Response: Thank you for suggestions! We are carefully revised the manuscript and check the grammar and syntax in manuscript that you pointed out.

COMMENTS FROM REVIEWER #2

Reviewer #2: Article Under Review: Tryptamine accumulation caused by deletion of MrMao-1 in Metarhizium genome significantly promotes insecticidal virulence

Summary:
In this study, the authors carefully characterize the high amount of tryptamine production by the specialist insect pathogenic fungus *Metarhizium acridum*, as compared to the generalist pathogen *Metarhizium robertsii*. They further convincingly link the production of tryptamine higher virulence by the specialist toward locusts, which is mediated by ROS accumulation via LmAhR. Through comparative genomics and transcriptomics, they discovered the tryptamine accumulation in the specialist is due to its lack of a monoamine oxidase gene required for tryptamine catabolism. This finding led to the production of a strain of *M. roberstii* wherein they deleted this gene. This mutant recapitulated the increased virulence and increased ROS seen in *M. acridum*, but irrespective of insect host. It seems they have uncovered a tryptamine-mediated path to enhanced virulence in pathogenic fungi with applicability in a wide range of insects. They rightly highlight that this method would not require transgenesis, as it could be accomplished through gene deletion.

Comments:
Q7) Ln 32: Throughout, is it more correct to say “inducing” instead of “regulating”?
Response: Thank you for suggestion. We corrected the “regulating” into “inducing”

Q8) Ln 46: I suggest referring to “MAA” as “MRO” throughout for consistency.

Response: We previously used MRO for the abbreviation of *Metarhizium roberstii*. However, Dr. Wang Chengshu, who supplied the fungus strain in this study, has nominated this strain as MAA because this strain is the same with *M. anisopliae*. Thus, we use the MAA to refer *M. roberstii* in this study. More information can be found in the manuscript (doi: 10.1073/pnas.1412662111) or website (https://mycocosm.jgi.doe.gov/Metan1/Metan1.home.html).

Q9) Ln 144: Please indicate which solvent you used to make your spore suspension.

Response: Thank you for the suggestion. We revised the “conidia of fungi” as “conidia of fungi that was suspended by the double distilled water containing 0.5%Tween-20”.

Q10) Ln 152-154: Is it more appropriate to say “up-regulated” instead of “expressed”, “triggered” and “activated”?

Response:

Response: These sentences described the total differentially expressed genes including up-regulated or down-regulated pattern in respond to MAA and MAC infections in Figure 1B. The results of up-regulated and down-regulated genes were indicated in Figure 1C and described in line 166-170. Therefore, we used the sentences as following to describe the results of Figure 1B: “The results showed that 359 genes were jointly induced in the locusts infected by MAC and MAA compared with the mock control. Approximately 800 genes were uniquely triggered by MAC, whereas only 110 unique genes were activated in response to MAA infection.”

Q11) Ln 155-156: Does this section of the Venn Diagram really show this? Did you limit your MAA vs. MAC comparisons to only MAC genes? Please make this more clear.

Response: Thank you for this comment. The Venn Diagram really showed this result as 162 genes (118+30+14). And we limit MAA vs. MAC comparisons to genes respond to fungal infections (including MAA and MAC infection). We revised as following: “By directly comparing the locusts infected by MAA and MAC, we found that 162 genes (sum of 118, 30, and 14) involved in response to fungal infection.”

Q12) Ln 185-188: These accession numbers do not pull up anything on NCBI, please check these.

Response: The accession numbers of MK125030, MK125031, MK125032, and MK140601 will be released on Dec 30, 2019. The attachment at the end of this letter is the original submission letters from NCBI and the accession hyperlink of these data. Now, I check the database and successfully access into these data.

Q13) Ln 252: Fascinating!

Response: We were also shocked by the higher level of tryptamine in MAC than MAA.

Q14) Ln 327: I recommend explicitly stating that this will be advantageous from a regulatory perspective.

Response: Good questions! We revised the sentence into “The findings showed that the high insecticidal virulence of MAC relayed on genes deletion instead of gene insertion. Because the deletion of enzyme genes in catabolic pathway leads to the accumulation of toxic metabolites for insects, the approaches for promoting insecticidal virulence of fungi can be used to develop highly efficient mycoinsecticides without introducing any exogenous gene.”

Q15) Ln 342-344: What does this sentence mean?

Response: We revised in the following: “Although narrow host ranges of MAC reduced its efficiency for harvesting tryptophan from insects, the tryptamine accumulation of MAC ensured its superiority of competence with MAA in promoting plants growth.”

Q16) Ln 345: Add a citation to justify “hasten the recycling of tryptophan from insects to plants”.

Response: Good suggestion! We added the article into references as reference 29: “S. W. Behie, P. M. Zelisko, M. J. Bidochka, Endophytic Insect-Parasitic Fungi Translocate Nitrogen Directly from Insects to Plants. *Science* 336, 6088 (2012) DOI: 10.1126/science.1222289”

Q17) Ln 355: This sentence seems to switch the order of AhR and ROS stress. Consider rewording.

Response: We revised the sentence into “activating AhR and increasing the ROS stress in fat body cells” according to your suggestion.

Q18) Ln 379: Your diagram suggests TDC, not Mao, is involved in the conversion tryptamine from tryptophan.

Response: We revised this ambiguous sentence into “the degradation of tryptamine at downstream of tryptophan catabolism is blocked in MAC due to the absence of *Mao* genes.”

Q19) Ln 382-388: Why would the generalist be better adapted to insects than the specialist? Also, why would the specialist be better adapted to plants? Generalists tend to be better plant symbionts than specialists. This section needs to be removed or rethought.

Response: Because plants preferred the organic nitrogen sources than inorganic nitrogen sources (Zhang et al., 2019; Näsholm et al., 2009), MAC is possibly better adapted to plants as symbiont due to the accumulate amines (organic nitrogen) than MAA that catabolized amines to ammonia (inorganic nitrogen). On the contrary, the production of ammonia benefits MAA for both induction and activation of fungal subtilisin proteases in the process of infection (Leger et al., 2009).

Therefore, we revised these sentences into: “Because plants preferred the organic nitrogen resources (Zhang et al., 2019; Näsholm et al., 2009), MAC is possibly better adapted to plants as symbiont due to the accumulation of amines than MAA that catabolized amines to ammonia. On the contrary, the production of ammonia benefits MAA for both induction and activation of fungal subtilisin proteases in the process of insect infection (Leger et al., 2009).”

We added the articles in references:

Ziliang Zhang, Yuanshuang Yuan, Qing Liu, *et al.* (2019) Plant nitrogen acquisition from inorganic and organic sources via root and mycelia pathways in ectomycorrhizal alpine forests. *Soil Biology and Biochemistry* 136:107517.

Torgny Näsholm, Knut Kielland, Ulrika Ganeteg. (2009) Uptake of organic nitrogen by plants. *New Phytologist*, 18*2:* 31-48*.*

Raymond J. St Leger, Judd O. Nelson, Steven E. Screen. (1999) The entomopathogenic fungus Metarhizium anisopliae alters ambient pH, allowing extracellular protease production and activity. *Microbiology*, 145: 2691-2699.

Q20) Ln 391: This statement about HGT is unfounded speculation, particularly considering how closely related Pochonia chlamydosporia and Metarhizium species are. Please remove.

Response: We removed this sentence according to your suggestion.

Q21) Ln 408-409: Please add a citation to back up this statement.

Response: We revised this statement by using the sentence “Fungus with tryptamine accumulation can also be used as bio-fertilizer to promote crop growth (Floch et al., 2003).”

We added the article into the reference: Gaétan Le Floch, Patrice Rey, Emile Benizri, *et al*., (2003) Impact of auxin-compounds produced by the antagonistic fungus *Pythium oligandrum* or the minor pathogen *Pythium* group F on plant growth. *Pla*nt and Soil, 257: 459-470.

Q22) Fig 1: Consider reporting percent survival on the y-axis instead of log rank throughout. Also, consider using colorblind-friendly coloration.
Response: We revised the y-axis of the figure and coloration according to your suggestions.

Suggested Revisions:
Q23) Ln 2: change “promotes” to “enhances”

Response: We revised the sentence according to your suggestions.

Q24) Ln 37: add space after “insects.”

Response: We revised the sentence according to your suggestions.

Q25) Ln 42: remove “traditional”

Response: We revised the sentence according to your suggestions.

Q26) Ln 43: change “is a fungus that” to “fungi”

Response: We revised the sentence according to your suggestions.

Q27) Ln 44: change “lives” to “live”; change “produces” to “can produce”
Response: We revised the sentence according to your suggestions.

Q28) Ln 50: add comma after “destruxins”
Response: We revised the sentence according to your suggestions.

Q29) Ln 63: change “these” to “the”
Response: We revised the sentence according to your suggestions.

Q30) Ln 64: change “safe” to “their safety”
Response: We revised the sentence according to your suggestions.

Q31) Ln 70: change “its” to “their”; change “it” to “they”; change “differentiates” to “differentiate”
Response: We revised the sentence according to your suggestions.

Q32) Ln 79: add comma after “hosts”
Response: We revised the sentence according to your suggestions.

Q33) Ln 82: change “germling” to “germlings”
Response: We revised the sentence according to your suggestions.

Q34) Ln 85: add “cuticular” before “penetration”; change “has” to “have”
Response: We revised these word according to your suggestion.

Q35) Ln 86: change “its” to “their”; remove “the” before “virulence”
Response: We revised the sentence according to your suggestions.

Q36) Ln 87: remove “of its pathogens”
Response: We revised the sentence according to your suggestions.

Q37) Ln 94: change “in charge of” to “required for”
Response: We revised the sentence according to your suggestions.

Q38) Ln 96: remove “enzymes”
Response: We revised the sentence according to your suggestions.

Q39) Ln 108: change “while” to “during”; swap “MAA” and “locusts”
Response: We revised the sentence according to your suggestions.

Q40) Ln 125: add “the” after “that”
Response: We revised the sentence according to your suggestions.

Q41) Ln 127: add “the” after “whereas”
Response: We revised the sentence according to your suggestions.

Q42) Ln 132: change “multi-copies” to “multiple copies”
Response: We revised the sentence according to your suggestions.

Q43) Ln 135: add “factor” after “virulence”
Response: We revised the sentence according to your suggestions.

Q44) Ln 151: change “each time of” to “their respective”
Response: We revised the sentence according to your suggestions.

Q45) Ln 175: change “part” to “components”
Response: We revised the sentence according to your suggestions.

Q46) Ln 188: remove comma
Response: We revised the sentence according to your suggestions.

Q47) Ln 203: remove “the”
Response: We revised the sentence according to your suggestions.

Q48) Ln 216: change “nuclei” to “nucleus”
Response: We revised “nuclei” to “nucleus” in the sentence, figure legends and figures.

Q49) Ln 235: change “missed” to “lacks the”
Response: We revised the sentence according to your suggestions.

Q50) Ln 236: remove “a”; capitalize “Quantification”; remove “analysis”; remove “the” after “in”
Response: We revised the sentence according to your suggestions.

Q51) Ln 335: change “destruxins” to “destruxin”
Response: We revised the sentence according to your suggestions.

Q52) Ln 337: change “plant” to “plants”
Response: We revised the sentence according to your suggestions.

Q53) Ln 346: change “plant” to “plants”
Response: We revised the sentence according to your suggestions.

Q54) Ln 352: change “confirmed” to “investigated”
Response: We revised the sentence according to your suggestions.

Q55) Ln 364: change “damages in” to “damage to”
Response: We revised the sentence according to your suggestions.

Q56) Ln 373: add period after “spp”
Response: We revised the sentence according to your suggestions.

Q57) Ln 408: replace “horizontal gene” with “transgene”
Response: We revised the sentence according to your suggestions.

Q58) Ln 420: remove “after”
Response: We revised the sentence according to your suggestions.

Q59) Ln 421: change “of” to “after”
Response: We revised the sentence according to your suggestions.

Q60) Ln 482: write O.C.T. in full
Response: We revised the sentence into “The locusts were quickly frozen by liquid nitrogen and then embedded with O.C.T. compound (O.C.T. is abbreviation of Optimal Cutting Temperature, Sakura Finetek, USA).”

Q61) Ln 532: change “Drug” to “Tryptamine”
Response: We used tryptamine and its antagonist SR1 (StemReginin 1) for analysis, therefore, we used word “Drug”.

Q62) Ln 607: change “P value” to “p-value”

Response: We revised the sentence according to your suggestions.

ORIGINAL LETTER FROM NCBI

From: gb-admin@ncbi.nlm.nih.gov

Data: 2018-11-05 23:23:20

To: tongxiwen@ioz.ac.cn

Subject: GenBank MK125030-MK125033

Dear GenBank Submitter:

Thank you for your direct submission of sequence data to GenBank. We

have provided GenBank accession numbers for your nucleotide sequences:

BankIt2164213 LOCMI00805	MK125030

BankIt2164214 LOCMI11675	MK125031

BankIt2164216 LOCMI16140	MK125032

BankIt2164219 LOCMI15907	MK125033

The GenBank accession numbers should appear in any publication that

reports or discusses these data, as it gives the community a unique label

with which they may retrieve your data from our on-line servers. You may

prepare and submit your manuscript before your accessions are released in

GenBank.

Submissions are not automatically deposited into GenBank after being

accessioned. Each sequence record is individually examined and processed

by the GenBank annotation staff to ensure that it is free of errors or

problems.

You have requested that your data are to be held confidential until:

Dec 30, 2019

They will not be released to the public database until this date,

or until the data or accession numbers appear in print, whichever

is first.

Since the flatfile record is a display format only and is not an editable

format of the data, do not make changes directly to a flatfile. For

complete information about different methods to update a sequence record,

see: <https://www.ncbi.nlm.nih.gov/Genbank/update.html>

Any inquiries about your submission should be sent to gb-admin@ncbi.nlm.nih.gov

For more information about the submission process or the available

submission tools, please contact GenBank User Support at

info@ncbi.nlm.nih.gov.

Please reply using the current Subject line.

Sincerely,

DeAnne Olsen Cravaritis, Ph.D.

The GenBank Direct Submission Staff

Bethesda, Maryland USA

ACCESSION HYPERLINK

MK125030: <https://www.ncbi.nlm.nih.gov/nuccore/MK125030>

MK125031: <https://www.ncbi.nlm.nih.gov/nuccore/MK125031>

MK125032: <https://www.ncbi.nlm.nih.gov/nuccore/MK125032>

MK125033: <https://www.ncbi.nlm.nih.gov/nuccore/MK125033>
