# Supplementary material for: Tryptamine accumulation caused by deletion of MrMao-1 in Metarhizium genome significantly enhances insecticidal virulence
Source: PLoS Genet. 2020 Apr 9;16(4):e1008675. doi: 10.1371/journal.pgen.1008675 (PMC7173932; doi:10.1371/journal.pgen.1008675)
Supplement: Attachment — Submitted filename: Response to reviewer mini R2.docx [file pgen.1008675.s008.docx]

COMMENTS FROM ASSOCIATE EDITOR

The authors have done a great job. The paper is very close. There are a few places that should be corrected, which I think makes the paper easier to read:

Q1) Line 88 I assume you mean specificity here, not specification

Q2) Line 90-91 sentence unclear: is it meant that 15 % of all putative genes are virulence genes?

Q3) Line 92 Do you mean ‘virulence genes’ here

Q4) Line 198 replace than with compared to

Q5) Lines 206-207: Rephrase. By comparing….., it was found that….

Q6) Line 214. …. To be between 1,585. – 20795 (put Cis in brackets)

Q7) Line 225: insect ‘in insects’ after AhR

Q8) Line 330 I think you mean ‘differences in’ here, rather than ‘diverse’?

Q9) Line 334 showS

Q10) Line 334 Relied instead of relayed

Q11) Line 334 gene not genes

Q12) Line 348 is expansive really meant, or expensive or widespread?

RESPONSE TO QUESTIONS

Q1) Line 88 I assume you mean specificity here, not specification

Response: Yes, we revised the sentence according to your suggestions to use the word “specificity”.

Q2) Line 90-91 sentence unclear: is it meant that 15 % of all putative genes are virulence genes?

Response: We cited the article (Reference 7 in manuscript) to identify the potential virulence-associated genes and found this study used a whole genome blast analysis against the pathogen-host interaction (PHI) gene database. Further, the article (Reference 10 in manuscript) showed the numbers of total putative proteins and PHI proteins in table 1 as following: *M. album* 1,308/8,427, 15.43%; *M. acridum* 1,628/9,849, 16.53%; *M. majus* 1,903/11,535, 16.5%; *M. guizhouense* 2,013/11,787, 17.08%; *M. brunneum* 1,868/10,689, 17.48%; *M.anisopliae* 1,874/10,891, 17.21%; *M. robersii* 1,956/11,689, 16.73%. Therefore, we revised sentence into “*Metarhizium* spp. have approximately 15% putative genes associated with virulence in the total proteins encoded in their genome.”

Q3) Line 92 Do you mean ‘virulence genes’ here

Response: Yes, we mean “virulence genes” here and revised sentence according to your suggestions.

Q4) Line 198 replace than with compared to

Response: We revised the sentence according to your suggestions.

Q5) Lines 206-207: Rephrase. By comparing….., it was found that….

Response: We revised the sentence according to your suggestions.

Q6) Line 214. …. To be between 1,585. – 20795 (put Cis in brackets)

Response: We revised the sentence according to your suggestions.

Q7) Line 225: insect ‘in insects’ after AhR

Response: We revised the sentence according to your suggestions.

Q8) Line 330 I think you mean ‘differences in’ here, rather than ‘diverse’?

Response: Yes, we mean “differences in” here and revised the sentence according to your suggestions.

Q9) Line 334 showS

Response: We revised the sentence according to your suggestions.

Q10) Line 334 Relied instead of relayed

Response: We revised the sentence according to your suggestions.

Q11) Line 334 gene not genes

Response: We revised the sentence according to your suggestions.

12) Line 348 is expansive really meant, or expensive or widespread?
Response: In organisms, *de novo* biosynthesis of tryptophan costs more energy than other amino acids. We mean “expensive” here and revised the sentence according to your suggestions.
